# Addressing the Need to Select Indicators of Adolescent Health: An Advisory Group's Reflections on the Journey

**DOI:** 10.1016/j.jadohealth.2024.02.004

**Published:** 2024-06

**Authors:** B. Jane Ferguson, Emmanuel Adebayo, Krishna Bose, Carolin Ekman, Charity R. Giyava, Ann Hagell, Sunil Mehra, Andrew D. Marsh

**Affiliations:** a Independent Consultancy, Tannay, Switzerland; b Institute of Child Health, College of Medicine, University of Ibadan, Ibadan, Nigeria; c Department of Population, Family and Reproductive Health, Johns Hopkins Bloomberg School of Public Health, Baltimore, Maryland; d Independent Consultancy, Geneva, Switzerland; e Women Deliver, Harare, Zimbabwe; f Association for Young People's Health, London, United Kingdom; g Mamta Health Institute for Mother and Child, New Delhi, India; h Department of Maternal, Newborn, Child, Adolescent Health and Ageing, World Health Organization, Geneva, Switzerland

**Keywords:** Adolescent health, Advisory groups, Indicators, Measurement

The Global Action for the Measurement of Adolescent health (GAMA) advisory group (AG) was formed in 2018 to meet a widely expressed need to recommend adolescent health indicators for use in all countries [1]. In line with similar efforts for other populations, the World Health Organization (WHO) made public calls for senior experts and young professionals to participate in a process that would provide advice on indicators related to adolescent health and identify measurement gaps in order to ensure that quality data are used to support programming and to relieve the reporting burden of countries. Of the 54 seniors and 113 young professionals responding to the calls, 12 seniors and four young persons were selected to join what became the GAMA AG. The selection considered measurement expertise, gender balance, and representation of all WHO regions in order to include various cultures, languages, and experiences. GAMA crafted its principal objective as: to propose a list of relevant, feasible, valid, and useful indicators to inform countries on what actions may be taken to assess and improve the health of adolescents, defined by WHO as those 10–19 years of age.

At the time of writing, GAMA has met nine times over four years. It will have met twice more by the publication of this supplement. Meetings most often included other participants and were most frequently virtual, due to COVID-19 restrictions. The AG contributed to three major activities during its initial phase, culminating with the selection of the draft list of indicators [[2], [3], [4]]. (Figure 1) This commentary presents a synthesis of the AG members' reflections on these activities. Our reflections are based on responses to an online survey of GAMA AG members about the process. These insights may provide useful context for understanding the overall effort and similar indicator prioritization efforts.

**Figure 1:**
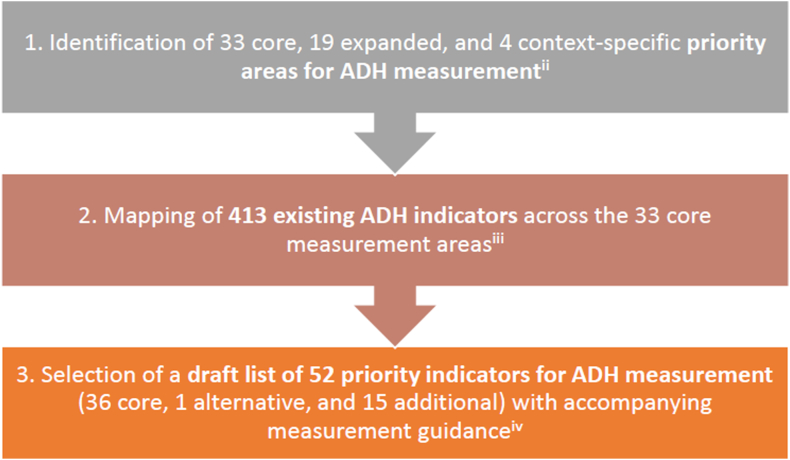
Major activities to select indicators of adolescent health prior to the survey of GAMA AG members.

GAMA AG members considered the first activity to be the most important one to achieve consensus, as this was when the parameters were set for the overall effort, i.e., the priority domains of adolescent health for measurement were identified. The third activity was also thought to be important due to the extensive inputs scrutinized.

A range of factors were considered to have been crucial to the successful identification of the indicators.

1. **Guidance by an effective and dedicated Secretariat,** diligent in summarizing and documenting the diverse information from which the indicators were identified and collated for review, helped to maintain momentum and focus to structure and move tasks steadily forward, while facilitating full participation.
2. **The continuous, transparent communication between the Secretariat and the AG** created a conducive environment for members to share, critique, question, and discuss, which in turn helped the group to arrive at informed and sagacious decisions.
3. **The appropriate use of diverse methodologies** to arrive at decisions. This included experiences from previous processes to identify and gain consensus on adolescent health indicators [5,6] and GAMA members' experiences with other indicator selection processes [7]. In addition, there were external inputs to the three activities, e.g., an online survey and the inclusion of representatives from four countries in one of the GAMA meetings. Internal input was gathered through discussions in plenary, combined with feedback from working groups, while also drawing on individuals' specific expertise for tasks in between the meetings, giving scope for more in-depth discussion and debate on particular indicators.
4. **The breadth of expertise among the AG members** was deemed to be a major strength in the critical examination and interpretation of the activities of the Secretariat.
5. **The young professionals** provided key connections with youth organizations, their own measurement experiences and perspectives, as well as ongoing reminders about the contexts of adolescents' lives. Their inclusion in the AG also provided opportunities for mentorship and capacity-building. Although several members wondered how the young professionals' involvement influenced the outcomes per se, ensuring young professionals active participation in all discussions and decisions crystallized a process that respected equity, inclusivity, and accountability.

The group also raised several challenges.

1. **The diversity among GAMA members in terms of their varied expertise in different measurement domains and experience with implementation in vastly dissimilar countries was both a strength and a barrier.** The group was intentionally heterogeneous, but this sometimes made it difficult to reach consensus on topics involving discussions among topic-specific experts and nonexperts. Fortunately, the iterative process of discussing specific topics in thematic subgroups and then presenting the outcomes to the full group was considered to have facilitated the necessary consensus. Interestingly, it was the technical diversity that was raised as a challenge and not, as might have been expected, the varied language and cultural backgrounds of the members.
2. **The task itself**—selecting a set of indicators that was both limited in number and broad in its coverage of the priority areas for the measurement of adolescent health in very disparate countries—inevitably required compromise. ‘Aspirational’ indicators, those that many members would like to see measured, were often revisited, with the final recognition of the need to align with those included in existing measurement systems.
3. **Although there was some country involvement in each of the major activities, some members expressed that more extensive involvement with countries would have improved the outcomes.** Regular inclusion of countries at different stages would have supported the identification of indicators with greater relevance and feasibility in real-life settings.

For many members, volunteering in the GAMA AG has been rooted in the recognition that measurement issues in countries are a primary barrier to improved programming for adolescents. They hoped that their expertise would lend perspective, credibility, and legitimacy to this scientific and political challenge. Despite this, there are concerns about the ultimate uptake of the recommended indicators and keen awareness of the hurdles ahead. Concerns include the acknowledgment that countries may not prioritize the measurement of adolescent health; the continued absence of appropriate age-disaggregation of routine data, even in the presence of electronic reporting systems; and the persistence of cultural barriers to collecting information about sensitive topics such as mental health, violence, and sexual and reproductive health, especially among the younger ages.

The journey that GAMA embarked on has resulted in a set of valid and robust indicators for adolescent health. Now, implementation of these indicators requires the GAMA recommendations to be accepted and used by political actors [8] and program staff in countries. UN partners can commence with regional orientation of stakeholders in countries, with “lead countries” providing perspectives on the context-specific feasibility and relevance of these indicators for their neighbors. The involvement of country stakeholders illustrated in the other papers of this supplement—related to acceptability, feasibility, harmonization, and application of the indicators—augur well for the future.

## Funding Sources

GAMA is funded by the 10.13039/100000865Bill and Melinda Gates Foundation [Investment IDs INV-001527 and INV-056035].
